# Thymidylate synthase expression identifies hepatocellular carcinoma vulnerable to fluoropyrimidine-based therapy

**DOI:** 10.1016/j.gendis.2026.102244

**Published:** 2026-05-17

**Authors:** Hyunji Choi, Yeeun Lee, Juyoung Lee, Jang-Yu Lee, Sangwon Byun, Kyunghee Noh, Kyung Chan Park, Wonbeak Yoo

**Affiliations:** a Genomic Medicine Research Center, Korea Research Institute of Bioscience and Biotechnology (KRIBB), Daejeon 34141, Republic of Korea; b Department of Functional Genomics, University of Science and Technology (UST), Daejeon 34113, Republic of Korea; c Bionanotechnology Research Center, Korea Research Institute of Bioscience and Biotechnology (KRIBB), Daejeon 34141, Republic of Korea; d Department of Nanobiotechnology, University of Science and Technology (UST), Daejeon 34113, Republic of Korea

Hepatocellular carcinoma (HCC) remains a global challenge with high mortality and heterogeneous treatment responses.^1^^,^^2^ While fluoropyrimidine-based chemotherapy, such as the FOLFOX regimen (a combination of fluorouracil, folinic acid, and oxaliplatin), is a clinical mainstay in Asian populations, the molecular determinants of its sensitivity remain poorly defined.^3^ Thymidylate synthase (TYMS), a key dTMP synthesis enzyme and primary 5-fluorouracil (5-FU) target, is linked to tumor progression and chemoresistance in various malignancies.^4^^,^^5^ However, the underlying regulatory mechanisms of TYMS and its functional implications in HCC pathogenesis remain insufficiently characterized, limiting its clinical and therapeutic application. To address these gaps, we employed an integrative multi-omics approach coupled with functional *in vitro* and *in vivo* validation.

To explore FOLFOX-associated molecular candidates in HCC, curated chemogenomic screening was performed using the STITCH database (Fig. 1A). Ten candidate proteins demonstrated drug–target connectivity, among which TYMS emerged as the most relevant hub (Fig. 1B). Consistently, analysis of the DepMap CRISPR-Cas9 database confirmed that HCC cell lines exhibited a strong genetic dependency on TYMS for proliferation and survival (Fig. S1). TYMS expression was significantly elevated in primary tumors compared with normal liver in TCGA-LIHC, and this pattern was consistently validated in independent GEO cohorts and paired tumor–normal analyses (Fig. 1C and Table S1). Spatial transcriptomics further confirmed region-specific up-regulation of TYMS within malignant hepatocyte niches (Fig. 1D). Increased TYMS protein abundance in HCC was supported by immunohistochemistry (HPA) and CPTAC proteomic datasets (Fig. 1E). Beyond its overexpression, analysis of the DepMap CRISPR-Cas9 dependency screen identified TYMS as a critical fitness gene in 24 HCC cell lines (median Chronos score < −0.5), confirming its essentiality for tumor cell survival (Fig. S1). Furthermore, TYMS expression was consistently associated with multiple clinicopathological factors, supporting its relevance as both a functional therapeutic target and a clinically meaningful biomarker.

**Figure 1:**
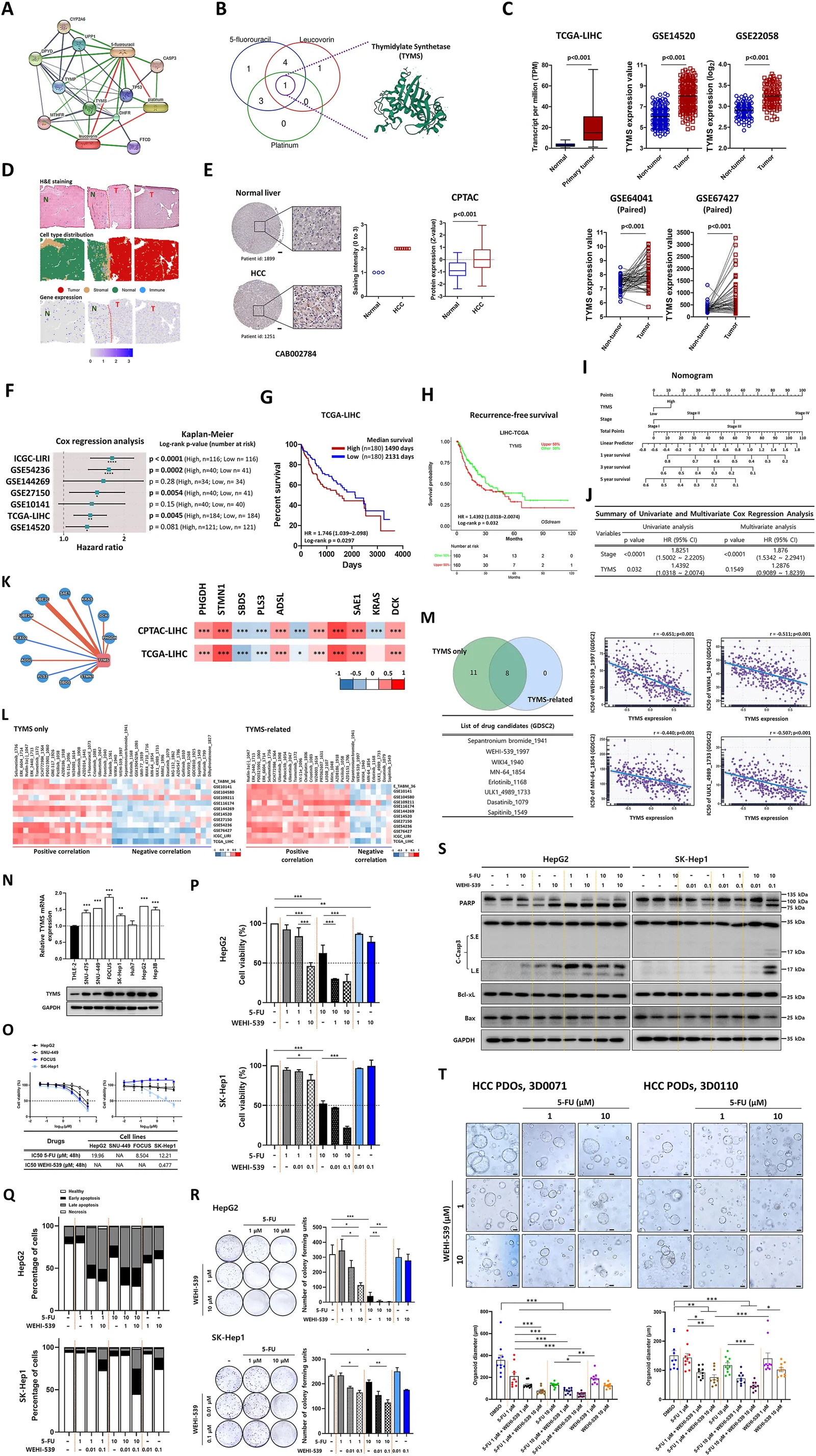
TYMS overexpression predicts poor outcome and drug sensitivity in hepatocellular carcinoma (HCC). **(A, B)** Network-based prediction of FOLFOX-associated protein targets using the STITCH database identified TYMS as a central hub with the highest interaction confidence score. **(C)** TYMS mRNA levels in tumor and normal tissues in the TCGA-LIHC dataset, including independent GEO datasets (GSE25097 and GSE76427). **(D)** Spatial transcriptomics showing TYMS distribution in tumor (T) and non-tumor (N) regions. **(E)** TYMS protein expression was assessed using immunohistochemistry and proteomics data from the HPA database, and further quantified in the CPTAC-LIHC proteomic dataset. **(F)** Overall survival analysis based on TYMS expression across seven public HCC cohorts; four datasets (ICGC-LIRI, GSE54236, GSE27150, TCGA-LIHC) showed significant prognostic value by Cox regression and log-rank test. **(G)** Kaplan–Meier survival curves stratified by TYMS expression in the TCGA-LIHC cohort, comparing high- versus low-expression groups. **(H)** Recurrence-free survival estimation using TYMS expression via a nomogram model in the TCGA-LIHC cohort. **(I)** Nomogram-based 1-, 3-, and 5-year survival prediction integrating TYMS and clinical staging variables. **(J)** Univariate and multivariate Cox regression analysis for TYMS expression and clinical stage in the TCGA-LIHC cohort. **(K)** A protein interaction network analysis of TYMS-associated genes in HCC revealed 11 candidate interactors (Spearman's *r* ≥ 0.3), and these associations were validated using the CPTAC-LIHC and TCGA-LIHC datasets. **(L)** Chemotherapeutic agents positively correlated with drug resistance in association with TYMS and related genes were identified using the GDSC2 dataset, whereas a subset of agents showed increased sensitivity relationships with TYMS and its associated gene network. **(M)** Distribution of sensitivity-associated drugs across TYMS-only and TYMS-shared subgroups, with heatmap-based identification of top-ranked candidates. Correlation between TYMS expression and drug sensitivity scores for WEHI-539, WIKI4, MN-64, and ULK1_4989. **(N)** The TYMS mRNA and protein levels in normal liver cell lines (THLE-2) and various HCC cell lines (SNU-475, SNU-449, FOCUS, SK-Hep1, HepG2, and Hep3B) were analyzed by quantitative reverse transcription PCR and Western blotting. **(O)** Dose–response curves and IC_50_ values for 5-fluorouracil (5-FU) and WEHI-539 in selected HCC cell lines. **(P)** Cell viability after single or combined treatment with 5-FU and WEHI-539 in HepG2 and SK-Hep1 cells. **(Q)** Flow cytometric analysis of apoptosis using Annexin V/PI staining after treatment with 5-FU, WEHI-539, or their combination in HepG2 and SK-Hep1 cells. **(R)** Colony formation assay showing reduced clonogenic potential following combination treatment compared with single-agent exposure. **(S)** Western blotting analysis of PARP cleavage, caspase-3 activation, and expression of Bcl-xL and Bax in cells treated with 5-FU, WEHI-539, or their combination. **(T)** Growth of HCC patient-derived organoids (PDOs) following drug treatment. 3D0071 and 3D0100 PDOs were seeded (1000 cells/10 μL Matrigel dome) and cultured for 3 days, followed by treatment with the indicated drug concentrations for an additional 3 days. PDO diameters were measured before cell viability assessment. For each group, 10 organoids were randomly selected for diameter quantification. Scale bar: 100 μm. Data represent mean ± standard deviation of three independent experiments. ∗*p* < 0.05, ∗∗*p* < 0.01, and ∗∗∗*p* < 0.001 versus control or as indicated.

Consistent with its essential role in tumor fitness, high TYMS expression was associated with significantly shorter overall survival across four independent cohorts (Fig. 1F). In the TCGA-LIHC dataset, tumors with high TYMS expression showed significantly shorter overall survival and recurrence-free survival compared with those with low expression (Fig. 1G and H). A predictive nomogram integrating TYMS and clinical stage showed enhanced prognostic resolution, although clinical stage remained the strongest independent variable in multivariate Cox analysis (Fig. 1I and J). These findings suggest that TYMS contributes to outcome stratification, particularly when integrated within prognostic modeling frameworks.

To characterize the molecular network associated with TYMS in LIHC, 16 candidates were identified by intersecting predicted interactors with positively correlated transcripts from TCGA (Fig. S2A and S2B). Principal component analysis (PCA) using this gene set effectively distinguished tumor from non-tumor samples (Fig. S2C). Most candidates showed significant mRNA up-regulation in both paired TCGA and independent GEO cohorts (Fig. S2D and S2E), which was further validated at the protein level via CPTAC and immunohistochemistry (Fig. S2F and S2G). This subset identifies a robust, multi-layer TYMS-linked molecular module in HCC. The prognostic relevance of these genes was subsequently assessed, revealing that elevated expression of a subset—including ATIC, DTYMK, NT5C, E2F1, and UBE2C—was associated with significantly poorer overall survival (Fig. S3A–S3C). Functional enrichment analyses indicated that these genes converged on pathways related to DNA replication, nucleotide synthesis, and cell-cycle regulation (Fig. S4A–S4C), which was further substantiated by GSEA Hallmark analysis (Table S2). Specifically, TYMS expression showed exceptional enrichment in E2F targets (NES = 2.98), G2M checkpoint (NES = 2.81), and DNA repair pathways (*p* < 1.0 × 10^−10^). These findings suggest that TYMS operates within a coordinated metabolic–proliferative network that orchestrates aggressive tumor growth and therapeutic resistance.

TYMS overexpression in HCC was investigated through promoter methylation and miRNA-mediated regulation. Promoter hypomethylation, observed across all clinical stages (Fig. S5A), correlated inversely with TYMS expression (Fig. S5B). Regarding post-transcriptional control, hsa-miR-192-5p and -215-5p were down-regulated in HCC tissues (Fig. S5D). Validation of hsa-miR-192-5p in a panel of HCC cell lines further confirmed its consistent and significant suppression compared with normal control cells (Fig. S5E). These candidates showed significant inverse correlations with TYMS levels (Fig. S5F). Functional enrichment linked these miRNAs to cancer-related pathways, including insulin and MAPK signaling (Fig. S5G). Collectively, these data suggest that both epigenetic hypomethylation and miRNA dysregulation drive TYMS up-regulation in HCC. Beyond its regulatory mechanisms, the clinical impact of TYMS on the tumor immune microenvironment was assessed. Single-cell transcriptomic profiling localized TYMS expression primarily to carcinoma cells, proliferating clusters, and scar-associated macrophages (Fig. S6A). Correlation analyses showed that TYMS expression was significantly associated with the infiltration of CD8^+^ T cells, macrophages, and myeloid-derived suppressor cells (Fig. S6B), while positively correlating with immune checkpoint genes such as CD276, CD274, and HAVCR2 (Fig. S6C). To further support these findings, immunohistochemical staining of TYMS, CD3, and CD11b in human HCC tissue microarrays revealed spatial patterns consistent with the inferred immune associations (Fig. S7). Multi-cohort cross-validation further confirmed robust positive correlations between TYMS and multiple immunomodulators, including PDCD1, CTLA4, and LAG3 (Fig. S8). Collectively, these findings suggest that TYMS is associated with an inflamed yet immunosuppressive tumor microenvironment, highlighting its potential relevance as a biomarker for immunotherapy response in HCC.

To explore the therapeutic implications of these findings, drug sensitivity and interaction analyses were performed. Eleven proteins showed potential interaction with TYMS, and most displayed positive expression correlations across CPTAC and TCGA datasets (Fig. 1K). Drug response profiling using the GDSC2 database identified 19 agents whose resistance signatures correlated positively with TYMS expression, while eight compounds exhibited increased sensitivity profiles (Fig. 1L). Among these, WEHI-539, WIKI4, MN-64, and ULK1_4989 demonstrated the strongest inverse correlation with TYMS expression (Fig. 1M). Molecular docking simulations revealed favorable binding affinities for WEHI-539, WIKI4, and MN-64, supporting their potential interaction with the TYMS protein (Fig. S9). These findings highlight WEHI-539, WIKI4, and MN-64 as candidate agents for repositioning in TYMS-overexpressing HCC. To validate the therapeutic relevance of repositioned agents, functional assays were performed in HCC cell lines with high TYMS expression compared with the normal liver cell line THLE-2 (Fig. 1N). WEHI-539 and WIKI4 were prioritized for experimental testing, with 5-FU included as a reference TYMS-targeting agent. Dose–response analyses demonstrated reduced viability with single-agent 5-FU and selective activity of WEHI-539, whereas WIKI4 showed minimal cytotoxicity (Fig. 1O; Fig. S10A). Combination treatment of 5-FU and WEHI-539 resulted in enhanced growth inhibition in HepG2, SK-Hep1, and Huh-7 cells (Fig. 1P; Fig. S10B), supported by increased apoptotic cell populations (Fig. 1Q; Fig. S10C–S10E), suppressed colony formation (Fig. 1R; Fig. S10F), and enhanced expression of apoptotic markers (Fig. 1S) compared with either agent alone. Consistent synergistic effects were also noted in SNU449 cells, while FOCUS cells remained relatively unresponsive (Fig. S10G). These synergistic effects were further validated in HCC patient-derived organoids, where the combination treatment significantly reduced organoid size and cell viability compared with monotherapies (Fig. 1T; Fig. S11). Together, these results indicate that co-targeting TYMS and BCL-xL using 5-FU and WEHI-539 may provide a synergistic therapeutic strategy for TYMS-high HCC.

In summary, this study identifies TYMS as a critical link between metabolic reprogramming and the immunosuppressive microenvironment in HCC. Our findings, validated through multi-cohort analyses and functional models—including liver organoids and patient-derived tissue microarrays—demonstrate that TYMS is a biologically grounded determinant of fluoropyrimidine sensitivity. These results establish TYMS as a multi-functional biomarker that, while the regulatory mechanisms of its expression and interaction with the tumor microenvironment warrant further study, extends beyond prognostic prediction and provides a mechanistic rationale for personalized therapeutic strategies in HCC.

## CRediT authorship contribution statement

**Hyunji Choi:** Writing – original draft, Visualization, Methodology, Conceptualization, Formal analysis, Funding acquisition. **Yeeun Lee:** Validation, Methodology, Data curation. **Juyoung Lee:** Resources, Data curation. **Jangyu Lee:** Data curation, Methodology, Visualization. **Sangwon Byun:** Writing – review & editing, Visualization. **Kyunghee Noh:** Data curation, Formal analysis, Funding acquisition, Investigation, Methodology, Resources, Supervision, Validation, Visualization, Writing – review & editing. **Kyung Chan Park:** Writing – review & editing, Supervision, Resources, Project administration, Investigation, Conceptualization. **Wonbeak Yoo:** Writing – review & editing, Supervision, Resources, Project administration, Investigation, Funding acquisition, Conceptualization.

## Data availability

The datasets used and/or analyzed during the current study are available from the corresponding author on reasonable request.

## Funding

This work was supported by grants from the National Research Foundation of Korea (NRF) funded by the Korea government (MSIT) (No. RS-2024-00338524, RS-2024-00338397, RS-2024-00451359), the KRIBB Research Initiative Program (No. KGM5192632, KGM1322612), and Seoul Clinical Laboratories (No. 2025AR01) in the Republic of Korea.

## Conflict of interests

The authors declared no competing interests.
