# Supplementary material for: Thymidylate synthase expression identifies hepatocellular carcinoma vulnerable to fluoropyrimidine-based therapy
Source: Genes Dis. 2026 May 17;14(3):102244. doi: 10.1016/j.gendis.2026.102244 (PMC13742270; doi:10.1016/j.gendis.2026.102244)
Supplement: Supplementary file 1 [file mmc1.docx]

**Thymidylate Synthase Expression Identifies Hepatocellular Carcinoma Vulnerable to Fluoropyrimidine-Based Therapy**

Hyunji Choi^1^, Yeeun Lee^1,2^, Juyoung Lee^1,2^, Jangyu Lee^3,4^, Sangwon Byun^1,2^, Kyunghee Noh^3,4*^, Kyung Chan Park^1,2,*^, Wonbeak Yoo^1,*^

**Address correspondence to:**

Kyunghee Noh., Bionanotechnology Research Center, Korea Research Institute of Bioscience and Biotechnology (KRIBB), Daejeon, 34141, Republic of Korea. Phone: +82-42-860-4442, E-mail: trollius@kribb.re.kr

Kyung Chan Park, Ph.D., Genomic Medicine Research Center, Korea Research Institute of Bioscience and Biotechnology, Daejeon, 34141, Republic of Korea. Phone: +82-42-879-8115, E-mail: kpark@kribb.re.kr

Wonbeak Yoo, Ph.D., Genomic Medicine Research Center, Korea Research Institute of Bioscience and Biotechnology, Daejeon, 34141, Republic of Korea. Phone: +82-42-879-8156, E-mail: wyoo@kribb.re.kr

**Supplementary Materials and methods**

**Materials and Methods**

Public data acquisition and processing

The chemical-protein interaction and prediction were obtained from STITCH database (1). To evaluate the functional essentiality of TYMS in HCC cell lines, CRISPR-Cas9 screening data were retrieved from the Dependency Map (DepMap) portal (https://depmap.org), and gene dependency scores were analyzed across various HCC cell lines. The gene expression of LIHC was obtained from samples of The Cancer Genome Atlas (TCGA) and The Gene Expression Omnibus (GEO, https://www.ncbi.nlm.nih.gov/geo/) dataset (GSE14520, GSE22058, GSE64041, and GSE67427). To further elucidate the overall survival (OS), Kaplan-Meier Plotter was conducted using TCGA-LIHC database with log-rank p-value and Hazard ratios (HR). The University of Alabama at Birmingham CANcer (UALCAN) database was used to analyze correlated gene expression, promoter methylation, protein expression (CPTAC, Clinical Proteomic Tumor Analysis Consortium), and clinicopathological parameters for TYMS gene in TCGA (2). Spatial transcriptomic analysis of TYMS was conducted using the “HCC4” dataset within the HCCDB portal (3). Expression patterns were visualized across tissue sections to characterize intra-tumoral spatial variability in HCC specimens. Besides, protein expression of TYMS and interacting proteins were validated with staining scores (0 to 3) in The Human Protein Atlas (HPA) dataset (https://www.proteinatlas.org/). According to the results of univariate and multivariate Cox Regression, DoSurvive (4) and OSdream database (5) was conducted whether TYMS has a risk of recurrence of LICH patients in the nomogram analysis. To define potential targets of TYMS, candidate miRNAs were predicted by miRabel database (6). We screened 16 miRNA candidates with 3’UTR binding based on excepting for miRNAs without values of PITA, miRanda, SVMicrO, and TargetScan in miRabel. The expression level, coefficient, KEGG pathway, and Disease Ontology was to analyze the Encyclopedia of RNA Interactomes (ENCORI) (7). The miRNA interactomes were identified by the predictive methods presented in the ENCORI program.

Cell culture

Cell culture were performed according to previously published protocols (8, 9) with minor modifications. Briefly, SNU-475, SNU-449, HepG2, and Huh7 cells were maintained in Roswell Park Memorial Institute 1640 (RPMI1640) medium, whereas FOCUS, Hep3B, and SK-Hep1 cells were cultured in Dulbecco’s modified Eagle’s medium (DMEM). THLE-2 cells were cultured in Bronchial Epithelial Cell Growth Medium (BEGM; #CC-3170), consisting of Bronchial Epithelial Cell Basal Medium (BEBM) supplemented with 5 ng/ml epidermal growth factor (EGF) and 70 ng/ml o-phosphorylethanolamine (Sigma Aldrich). All cell lines were incubated in growth medium supplemented with 10% fetal bovine serum (Gibco, Grand Island, NY, USA) and 1% penicillin–streptomycin (Gibco, USA) at 37 °C in a humidified atmosphere with 5% CO₂.

Liver cancer organoid culture and drug treatment

3D0071 and 3D0110 was established from liver cancer. Organoid were obtained from KCTC(Korean Cellection for Type Cultures). The Organoids were incubated in a 37 ℃ and 5% CO_2_ culture incubator. The culture medium consisted of 1× advanced Dulbecco’s Modified Eagle Medium/Ham’s F-12), 1 × GlutaMAX supplement, HEPES (10 mM), 1 × N2 supplement, N-acetylcysteine (1 mM), [Leu15]-GastrinI (10 nM), human EGF (50 ng/mL), human HGF (25 ng/mL), 1 × B27 supplement, A83-01 (5 μM), nicotinamide (10 mM), Forskolin (10 μM), human R-spondin (1μg/ml), human FGF10 (100 ng/ml), and 1 × Antibiotic-Antimycotic (GIBCO, Cat# 15240062). The culture medium was refreshed every two to three days depending on growth rate. To passage the organoids, the BME dome was broken up using TrypLE Express (GIBCO) and pipetted into a 15 mL conical tube. The dome was further disrupted by forceful pipetting, then incubated at 37 °C for 10 min. Subsequently, three volumes of basal medium containing 10% FBS were added to the suspension. The domes were centrifuged at 10 ×g for 3 min, and the supernatant was removed. Once BME was removed, the dissociated organoid pellet was resuspended in basal medium + Matrigel (1:3) and reseeded at 1:3-1:4 rations to allow the formation of new organoids. The new dome (50 μL) was seeded in 6 well plate and incubated at 37 °C for 10 min. After the dome had solidified, 3 mL of the culture medium was gently added to the each well to cover the domes and organoids were incubated in a 37 ℃ and 5% CO_2_ incubator. For drug treatment, single organoids (1,000 cells per dome in 10 μL) were seeded into 48-well plates and allowed to recover for 3 days. Once the organoids reached a diameter of approximately 50–100 μm, they were treated with varying concentrations of 5-fluorouracil (5-FU) and WEHI-539 for an additional 3 days. Organoid size was monitored by measuring the diameter under a microscope. Cell viability was subsequently assessed using the CellTiter-Glo® 3D assay according to the manufacturer’s instructions.

RNA isolation and quantitative real-time PCR (qRT-PCR)

Total cellular RNA was isolated using TRIzol reagent (Invitrogen, USA) following the manufacturer’s instructions. Complementary DNA (cDNA) was synthesized from 1 μg of RNA with the RevertAid First Strand cDNA Synthesis Kit (Thermo Fisher Scientific, USA). Quantitative real-time PCR was carried out using SYBR Green master mix (Thermo Fisher Scientific, USA) on a standard thermal cycler. Primer sequences were designed using Primer3 software. The following primers were used for amplification of target mRNAs: GAPDH (forward 5′-ACCATCTTCCAGGAGCGAGA-3′, reverse 5′-AGTGATGGCATGGACTGTGG-3′) and TYMS (forward 5′-ATCTTCCTCTGATGGCGCTG-3′, reverse 5′-AAAGGTCTGGGTTCTCGCTG-3′). Gene expression levels were normalized to GAPDH, and relative expression was determined using the 2^−^ΔΔCt^ method (10). For miRNA expression analysis, total RNA was extracted using the miRNeasy Kit (Qiagen, Netherlands). Reverse transcription was performed with the Verso cDNA Synthesis Kit (Thermo Fisher Scientific) with specific stem-loop RT primers: 5′-GTCGTATCCAGTGCAGGGTCCGAGGTATTCGCACTGGATACGACGGCTGTC-3′ for miR-192-5p and 5′-CTCAACTGGTGTCGTGGAGTCGGCAATTCAGTTGAGAAAAATATG-3′ for U6. Quantitative real-time PCR was carried out using SYBR Green master mix (Thermo Fisher Scientific) on an Applied Biosystems or Agilent system. The following primer sets were used for amplification: GAPDH (forward 5′-ACCATCTTCCAGGAGCGAGA-3′, reverse 5′-AGTGATGGCATGGACTGTGG-3′), TYMS (forward 5′-ATCTTCCTCTGATGGCGCTG-3′, reverse 5′-AAAGGTCTGGGTTCTCGCTG-3′), miR-192-5p (forward 5′-CTGACCTATGAATTGACAGCC-3′, reverse 5′-GTGCAGGGTCCGAGGT-3′), and U6 (forward 5′-CTCGCTTCGGCAGCACA-3′, reverse 5′-AACGCTTCACGAATTTGCGT-3′). Gene and miRNA expression levels were normalized to GAPDH and U6, respectively, and relative quantification was calculated using the 2^−^ΔΔCt^ method.

Drug sensitivity assay

For drug sensitivity assays, cells were seeded in 96-well plates at the following densities: HepG2, Huh-7 and SNU-475, 5 × 10³; SNU-449; 7 × 10³; FOCUS, SK-Hep1 and Hep3B 3 × 10³ cells per well, and incubated at 37 °C. After cell attachment, cells were treated with 5-fluorouracil (5-FU; HY-90006, MCE, Wuhan, China), WEHI-539 (HY-15607, MCE, Wuhan, China) and WIKI4 (L1100, Selleckchem, Houston, TX, USA) at serial threefold dilutions. The treatment concentration ranged from 0.04 to 30 μM for 5-FU and 0.04 to 10 μM for WEHI-539 and WIKI4, and ther cells were incubated for 48 hours. Cell viability was measured using the WST-1 reagent (EasyCytox, Korea), and absorbance was recorded at 450 nm using a microplate reader. Dose-response curves were generated, and IC50 values were calculated using GraphPad Prism 10.2. Based on the calculated IC50 values, appropriate concentrations were selected for single-agent and combination drug treatments in subsequent experiments. HepG2 and SK-Hep1 cells were treated with 5-fluorouracil (5-FU) at concentrations of 1 and 10 µM. For WEHI-539 treatment, doses of 1 and 10 µM were applied to HepG2 cells, while lower concentrations of 0.01 and 0.1 µM were used for SK-Hep1 cells. In Huh-7 cells, a fixed dose of 5 µM 5-FU was administered in combination with 1 and 10 µM of WEHI-539 to evaluate their synergistic effects. All experiments were performed in triplicate.

Colony formation assay

To assess colony formation potential, HepG2 cells were seeded in 6-well plates at a density of 3 × 10³ cells per well, while SK-Hep1 and Huh-7 cells were seeded at 1 × 10³ cells per well. After cell attachment, the cells were treated with individual drugs or drug combinations. After 48 hours of treatment, the culture medium was replaced with fresh medium and subsequently renewed every 2 days during the 7-day incubation period. At the end of the incubation, the cells were washed twice with PBS and stained with 0.5% crystal violet for 30 minutes at room temperature. The plates were then washed with PBS, and colonies were counted and imaged for quantification.

Western blot analysis

For western blot analysis, Cells were seeded in 6-well plates and allowed to reach 70–80% confluence. After cell attachment, the cells were treated with individual drugs or drug combinations for 24 or 48 hours. Cells were then harvested, washed with cold PBS, and lysed on ice for 30 minutes using RIPA buffer (150 mM NaCl, 1% NP-40, 0.5% sodium deoxycholate, 0.1% SDS, and 25 mM Tris-HCl, pH 7.4) supplemented with a protease and phosphatase inhibitor cocktail (Roche, Switzerland). Protein concentrations were determined using the BCA protein assay kit (Thermo Fisher Scientific, USA). Equal amounts of total protein were separated by SDS–PAGE and electro transferred onto polyvinylidene difluoride (PVDF) membranes. After blocking with 5% skim milk in PBST (0.1% Tween-20) for 1 h, membranes were incubated overnight at 4 °C with primary antibodies against TYMS (#9045), PARP (#9542), caspase-3 (#9662), Bcl-xL (#2762), and Bcl-2 (#15071) (all from Cell Signaling Technology, USA), Bax (sc-7480, Santa Cruz Biotechnology, USA), and GAPDH (GB1002, Servicebio, China) as a loading control. After washing, the membranes were incubated with HRP-conjugated secondary antibodies for 1 hour at room temperature: goat anti-rabbit IgG (H+L), HRP (Cat. No. 31460, Invitrogen, USA) and goat anti-mouse IgG (H+L), HRP (ab6789, Abcam, UK). Immunoreactive bands were visualized using the SuperSignal™ West Pico PLUS chemiluminescent substrate (Thermo Fisher Scientific, USA) and imaged with a ChemiDoc™ Imaging System (Bio-Rad, USA).

Immunofluorescence (IF) analysis

Human tissue microarray staining was performed on a commercial tissue array (#CSN; SuperBioChips, Korea). Tissue sections were deparaffinized in xylene and rehydrated through a graded ethanol series. For antigen retrieval, the section was immersed in 0.1 M citrate buffer and heated for 20 minutes. Blocking was performed using the Mouse on Mouse (M.O.M.) Immunodetection Kit (#BMK-2202; Vector Laboratories, USA) according to the manufacturer’s instructions to minimize non-specific binding. Sections were incubated with primary antibodies diluted in blocking buffer at 4°C overnight. After washing, sections were incubated with appropriate fluorescent-conjugated secondary antibodies for 1 hour and then with DAPI solution for 10 minutes at room temperature in the dark. Sections were mounted using a fluorescence mounting medium, and images were acquired using confocal microscopy (Leica, Germany). The following primary antibodies were used: TYMS (#9045T) and CD11b (#63965T) from Cell Signaling Technology, and CD3 (#344803) from BioLegend.

Flow Cytometry Analysis

Apoptosis was assessed using the FITC Annexin V apoptosis detection kit (BD Biosciences, Bedford, MA, USA) according to the manufacturer’s protocol. Briefly, cells were seeded in 6-well plates at the following densities: HepG2, 3 × 10⁵ cells per well; SK-Hep1 and Huh-7, 2 × 10^5^ cells per well, and incubated at 37 °C. After cell attachment, cells were treated with individual drugs or drug combinations for 24 or 48 hours. Following treatment, cells were washed with PBS, harvested by centrifugation, and incubated with Annexin V, propidium iodide (PI), and 100 µL of annexin-binding buffer for 15 minutes at room temperature in the dark. The reaction was terminated by adding 100 µL of annexin-binding buffer per tube, and apoptosis was analyzed using an Attune NxT flow cytometer (Thermo Fisher Scientific, USA).

Biological function annotation by multi-omics

The 16 genes with the strongest correlation with TYMS were identified TCGA-LIHC (positive correlation; 5387 genes) and Pathway Commons (interaction; 24 genes), and this allowed the Principal component analysis (PCA) in GEPIA2 database (11). To further elucidate meaning of functions and pathways, ontological analysis (GO Biological Process 2023), pathway analysis (KEGG 2021 and Reactome 2022) for 16 selected genes forming a cluster with TYMS was performed using Enrichr (12).

Estimation of immune cell infiltration in tumor microenvironment

The Human Liver Browser was employed to investigate the single-cell transcriptomic profiles between non-malignant cells and malignant cells in LIHC (13). The Tumor Immune Estimation Resource database (TIMER 2.0) was applied to analyze the immune cell infiltration of CD8+ T cells, CD4+ T cells, macrophages, B cells, natural killer (NK) cells, Regulatory T (Treg) cells, myeloid-derived suppressor cells (MDSC), and cancer associated fibroblasts (CAFs) from TCGA-LIHC (14). All correlation strength were estimated statistical significance with automatically calculated based on the online after the purity-adjusted Spearman’s Rho.

Comprehensive analysis of TYMS-targeted drug repurposing and its correlation with immune checkpoints in HCC

To determine the drug repurposing of TYMS, Protein Interaction Network Analysis (PINA. v3.0) was conducted to select TYMS interacting proteins with CPTAC-LIHC and TCGA-LIHC. Using the BEST tool (15), we analyzed drug sensitivity correlations in the Genomics of Drug Sensitivity in Cancer (GDSC2) for each gene and conducted GSEA Hallmark and immunomodulation analyses, subsequently identifying common agents as potential repurposing drugs for TYMS and its interacting proteins. Molecular docking studies were carried out using the AutoDock Vina docking engine implemented in SwissDock (https://www.swissdock.ch/). Candidate drugs with the lowest binding free energy were considered to represent the most favorable interactions. Additionally, list of immune checkpoint molecules was from the published literature (16), and analysis of association between immune checkpoint molecules and TYMS expression was performed at mRNA level in TCGA-LIHC.

Statistical analysis

Cell viability and cytotoxicity assays were conducted to evaluate the effects of 5-FU, WEHI-539, and their combination. Statistical analyses were performed using two-way analysis of variance (ANOVA) with 5-FU and WEHI-539 as independent variables to assess main and interaction effects. When significant differences were identified, Tukey’s multiple-comparison test was applied for post hoc analysis, and multiplicity-adjusted P values were reported. All analyses were carried out using GraphPad Prism (version 10). Correlation analyses, where applicable, were performed using Spearman’s rank correlation. A P value < 0.05 was considered statistically significant.

**Table S1.** Clinicopathological features in HCC patients from TCGA-LIHC cohort

|  | **Expression of TYMS Transcript per million (Median value)** | **p-value**  **(* vs. normal)** |
| --- | --- | --- |
| **Sex** |  |  |
| Normal (n=50) | 0.37 - 7.969 (2.789) |  |
| Tumor_Male (n=245) | 1.492 - 68.351 (13.838) | < 0.001* |
| Tumor_Female (n=117) | 1.37 - 82.817 (16.034) | < 0.001* |
| **Age** |  |  |
| Normal (n=50) | 0.37 - 7.969 (2.789) |  |
| Tumor (age 21 - 40, n=27) | 1.344 - 68.85 (16.153) | < 0.001* |
| Tumor (age 41 - 60, n=140) | 1.492 - 82.817 (17.091) | < 0.001* |
| Tumor (age 61 - 80, n=181) | 1.137 - 58.526 (13.042) | < 0.001* |
| Tumor (age > 80, n=10) | 3.221 - 34.168 (17.739) | 0.038*, 0.005** |
| **** vs. Age 41 - 60** |  |  |
| **Race** |  |  |
| Normal (n=50) | 0.37 - 7.969 (2.789) |  |
| Caucasian (n=177) | 1.137 - 64.191 (13.042) | < 0.001* |
| African-american (n=17) | 1.864 - 77.47 (15.079) | 0.003* |
| Asian (n=157) | 1.492 - 82.612 (17.269) | < 0.001*, 0.006** |
| **** vs. Caucasian** |  |  |
| **Tumor stage** |  |  |
| Normal (n=50) | 0.37 - 7.969 (2.789) |  |
| Stage I (n=168) | 1.492 - 49.639 (11.252) | < 0.001* |
| Stage II (n=84) | 1.137 - 82.817 (17.772) | < 0.001*, 0.007** |
| Stage III (n=82) | 1.344 - 116.271 (26.057) | < 0.001*, < 0.001*** |
| Stage IV (n=6) | 6.687 - 15.7 (7.275) | n.s |
| **** vs. Stage I; *** vs. Stage I** |  |  |
| **Tumor grade** |  |  |
| Normal (n=50) | 0.37 - 7.969 (2.789) |  |
| Grade I (n=54) | 2.441 - 44.076 (8.366) | < 0.001* |
| Grade II (n=173) | 1.137 - 60.967 (12.247) | < 0.001* |
| Grade III (n=118) | 2.14 - 98.33 (20.399) | < 0.001*, 0.048**, 0.002*** |
| Grade IV (n=12) | 2.768 - 63.199 (22.174) | 0.002* |
| **** vs. Grade I; *** vs. Grade II** |  |  |
| **Histological subtype** |  |  |
| Normal (n=50) | 0.37 - 10.996 (3.003) |  |
| Hepatocellular carcinoma (HCC, n=361) | 1.137 - 75.743 (14.941) | < 0.001* |
| Fibrolamellar carcinoma (FC, n=3) | 5.489 - 35.84 (28.653) | 0.020* |
| Hepatocholangiocarcinoma (Mixed, n=7) | 1.521 - 8.659 (3.958) | n.s, 0.001**, 0.023*** |
| **** vs. HCC; *** vs. FC** |  |  |

**Table S2. GSEA Hallmark analysis of the TYMS gene in TCGA-LIHC**

| **Description** | **Enrichment**  **Score** | **NES** | **p-value** | **Adjusted**  **p-value** | **q-values** |
| --- | --- | --- | --- | --- | --- |
| E2f targets | 0.8493444 | 2.982211554 | 1.00E-10 | 6.50E-10 | 4.21E-10 |
| G2m checkpoint | 0.800771285 | 2.812315074 | 1.00E-10 | 6.50E-10 | 4.21E-10 |
| Myc targets v1 | 0.74989178 | 2.633014277 | 1.00E-10 | 6.50E-10 | 4.21E-10 |
| Dna repair | 0.661095223 | 2.296006727 | 1.00E-10 | 6.50E-10 | 4.21E-10 |
| Mtorc1 signaling | 0.600320621 | 2.107841169 | 1.00E-10 | 6.50E-10 | 4.21E-10 |
| Myc targets v2 | 0.655335689 | 2.098826079 | 1.46E-08 | 7.10E-08 | 4.60E-08 |
| Mitotic spindle | 0.597078907 | 2.096945833 | 1.00E-10 | 6.50E-10 | 4.21E-10 |
| Unfolded protein response | 0.565299159 | 1.921259592 | 6.09E-09 | 3.39E-08 | 2.20E-08 |
| Protein secretion | 0.557337527 | 1.858456477 | 2.20E-07 | 9.54E-07 | 6.18E-07 |
| Oxidative phosphorylation | 0.467060514 | 1.639939302 | 2.16E-06 | 7.65E-06 | 4.96E-06 |
| Pi3k akt mtor signaling | 0.48492403 | 1.630370696 | 1.79E-04 | 5.36E-04 | 3.47E-04 |
| Glycolysis | 0.452367687 | 1.588349959 | 1.47E-05 | 4.78E-05 | 3.10E-05 |
| Spermatogenesis | 0.442157518 | 1.522155411 | 1.81E-03 | 4.69E-03 | 3.04E-03 |
| Uv response up | 0.428520837 | 1.490274998 | 6.15E-04 | 1.71E-03 | 1.11E-03 |
| Bile acid metabolism | -0.37518196 | -2.044039783 | 5.71E-07 | 2.23E-06 | 1.44E-06 |

Statistically significant hallmarks were selected based on a cut-off of q-value < 0.05.

**Figure S1.** Functional essentiality of TYMS in HCC cell lines based on DepMap CRISPR-Cas9 screening. Distribution of TYMS gene effect scores (Chronos) across 24 liver cancer cell lines from the DepMap Public 25Q3 (CRISPR-Cas9) dataset. The density plot (left) illustrates the distribution of scores, where 0 and -1 represent the median effect of non-essential and common essential genes, respectively. The accompanying table (right) summarizes key metrics: a median Chronos score of -0.87, maximum dependency observed in JHH6 (-2.96), and minimum dependency in KMCH1 (0.02). Approximately 52% of the tested lines are classified as essential, defined by a Chronos score < -0.5.

**Figure S2.** Identification and expression analysis of TYMS-related genes in HCC. (A) Interacting genes of TYMS predicted from the PANTHER database. (B) Overlapping gene set (n=16) between TYMS-interacting genes (n=24, PANTHER) and positively correlated genes in TCGA-LIHC dataset (n=5,387). (C) Principal component analysis (PCA) of the 16 TYMS-related genes distinguishing tumor from control samples in TCGA-LIHC. (D) mRNA expression levels of 16 TYMS-related genes in HCC tumors vs. normal tissues in TCGA-LIHC. (E) Paired tumor and adjacent non-tumor comparison of TYMS-related gene expression in GEO database (GSE64041). (F) Protein expression patterns of TYMS-associated genes in CPTAC-LIHC dataset. (G) Immunohistochemical staining scores from the Human Protein Atlas (HPA) database comparing protein levels in normal liver and LIHC tissues for selected genes.

**Figure S3.** Prognostic assessment and classification based on TYMS-related genes in TCGA-LIHC. (A) Kaplan–Meier analysis of overall survival according to mRNA expression levels of 14 TYMS-associated genes in the TCGA-LIHC cohort. (B) Univariate Cox regression analysis evaluating the impact of clinical stage and gene expression on overall survival for each gene. (C) Combined stratification of patients into four groups based on median expression of TYMS and 10 selected genes; survival curves and statistical results from log-rank (LR) and log-rank trend (LRT) tests are shown.

**Figure S4.** Functional enrichment of TYMS-related gene set based on (A) GO Biological Process, (B) KEGG, and (C) Reactome pathway analyses. GO terms include DNA biosynthetic process, cell cycle phase transition, and nucleotide metabolism; KEGG pathways involve pyrimidine and purine metabolism, and drug metabolism; Reactome pathways cover nucleotide interconversion, nucleotide metabolism, and cell cycle regulation.

**Figure S5.** Promoter methylation and miRNA-mediated regulation as upstream mechanisms of TYMS in HCC. (A) TYMS promoter methylation levels in normal and LIHC tissues across tumor stages and histological grades. (B) Correlation analysis between TYMS promoter methylation and TYMS mRNA expression in LIHC. (C) Predicted TYMS-targeting miRNAs identified using miRable. (D) Differential expression of candidate miRNAs in normal vs. HCC tissue samples. (E) Relative expression levels of hsa-miR-195-5p in a normal liver cell line (THLE2) and various HCC cell lines. (F) Correlation between TYMS mRNA expression and selected miRNAs (hsa-miR-192-5p, hsa-miR-215-5p). (G) KEGG pathway enrichment for hsa-miR-192-5p and hsa-miR-215-5p. (H) Disease Ontology enrichment results for hsa-miR-192-5p and hsa-miR-215-5p. *P < 0.05, **P < 0.01, ***P < 0.001, ****P < 0.0001 versus as indicated.

**Figure S6.** Cell-type-specific TYMS expression and its correlation with immune infiltration in HCC. (A) Cell type–specific distribution of TYMS expression in HCC tissue from the single-cell dataset, including carcinoma cells, proliferating cells, scar-associated macrophages (SAMs), hepatocytes, pericytes, and vascular smooth muscle cells (vSMCs). (B) Correlation between TYMS expression and immune cell infiltration levels (CD8⁺ T cells, CD4⁺ T cells, macrophages, myeloid-derived suppressor cells [MDSCs], cancer-associated fibroblasts [CAFs], and natural killer [NK] cells) based on relative immune cell enrichment based on transcriptomic signatures. (C) Correlation analysis of TYMS expression with immune checkpoint–related genes in tumor versus normal tissues from TCGA-LIHC dataset. *P < 0.05, **P < 0.01, ***P < 0.001.

**Figure S7.** Representative immunofluorescence and H&E images of human HCC tissue microarrays. Multiplex immunofluorescence (IF) staining was performed on human hepatocellular carcinoma (HCC) tissue microarrays (TMA). The panels show representative images from four different patients (#5, #32, #28, and #18). CD11b (green) or CD3 (green), TYMS (red), and DAPI (blue, for nuclear staining) are shown individually and as merged images to visualize their spatial distribution and co-localization. Corresponding H&E (Hematoxylin and Eosin) stained sections provided by the manufacturer (SuperBioChips) are displayed on the right to show the histological features of the corresponding patient samples. Images were captured using confocal microscopy.

**Figure S8.** Association between TYMS expression and various classes of immunomodulators across multiple HCC cohorts. Heatmaps showing Spearman correlations between TYMS expression and various immunomodulators across multiple HCC cohorts (TCGA-LIHC, ICGC-LIRI, and GSE datasets). Immunomodulators are categorized into five functional groups: antigen presentation, immunoinhibitors, immunostimulators, chemokines, and receptors. The color scale indicates Spearman correlation coefficients, with red and blue representing positive and negative correlations, respectively.

**Figure S9. Molecular docking simulation of candidate compounds with the target protein using SwissDock.** Binding modes of candidate compounds (WEHI-539_1997, WIKI4_1940, and MN-64_1854) were predicted using the SwissDock engine implemented with AutoDock Vina. (Left) 2D chemical structures of the compounds: The tables below represent the calculated binding free energy (kcal/mol), where Model 1 denotes the pose with the lowest binding energy.

**Figure S10.** Drug Sensitivity and Apoptotic Response to 5-FU, WEHI-539, and WIKI4 in HCC Cells. (A) Dose–response curves and values for 5-FU, WEHI-539, and WIKI4 in HCC cell lines. (B) Cell viability of Huh-7 cells after single or combined treatment with 5-FU and WEHI-539. (C–D) Flow cytometric analysis of apoptosis via Annexin V/PI staining in Huh-7 cells following indicated treatments. (E) Apoptosis analysis of HepG2 and SK-Hep1 cells via Annexin V/PI staining after indicated treatments. (F) Colony formation assay of Huh-7 cells following single or combined treatment with 5-FU and WEHI-539. (G) Cell viability of SNU449 and FOCUS cells after single or combined treatment with 5-FU and WEHI-539. Data represent mean ± SD of three independent experiments. *P < 0.05, **P < 0.01, ***P < 0.001 versus control or as indicated.

**Figure S11.** Viability of HCC patient-derived organoids (PDOs) following drug treatment. HCC PDOs, 3D0071 (left graph) and 3D0100 (right graph), were seeded at a density of 1,000 cells per 10 μL Matrigel dome and cultured for 3 days. The organoids were then treated with the indicated drug concentrations for an additional 3 days. Cell viability was assessed using the CellTiter-Glo® 3D assay; luminescence was measured after 10 minutes of shaking. Data are presented as the mean ± SD of three independent experiments. *p < 0.05, **p < 0.01, and ***p < 0.001 vs. control or as indicated.

**References**

1.	Szklarczyk D, Santos A, von Mering C, Jensen LJ, Bork P, Kuhn M. STITCH 5: augmenting protein-chemical interaction networks with tissue and affinity data. Nucleic Acids Res. 2016;44(D1):D380-4.

2.	Chandrashekar DS, Karthikeyan SK, Korla PK, Patel H, Shovon AR, Athar M, et al. UALCAN: An update to the integrated cancer data analysis platform. Neoplasia. 2022;25:18-27.

3.	Jiang Z, Wu Y, Miao Y, Deng K, Yang F, Xu S, et al. HCCDB v2.0: Decompose Expression Variations by Single-cell RNA-seq and Spatial Transcriptomics in HCC. Genomics Proteomics Bioinformatics. 2024;22(1).

4.	Wu HW, Wu JD, Yeh YP, Wu TH, Chao CH, Wang W, et al. DoSurvive: A webtool for investigating the prognostic power of a single or combined cancer biomarker. iScience. 2023;26(8):107269.

5.	Li H, Wang Q, Liu Y, Chen L, Zhao Q, Xie L, et al. OSdream: An online survival and differential analysis tool of recurrence and metastasis of pan-cancers. Genes Dis. 2025;12(4):101446.

6.	Quillet A, Saad C, Ferry G, Anouar Y, Vergne N, Lecroq T, et al. Improving Bioinformatics Prediction of microRNA Targets by Ranks Aggregation. Front Genet. 2019;10:1330.

7.	Li JH, Liu S, Zhou H, Qu LH, Yang JH. starBase v2.0: decoding miRNA-ceRNA, miRNA-ncRNA and protein-RNA interaction networks from large-scale CLIP-Seq data. Nucleic Acids Res. 2014;42(Database issue):D92-7.

8.	Yoo W, Kim AK, Kook HU, Noh K. Comprehensive analysis on clinical significance and therapeutic targets of LDL receptor related protein 11 (LRP11) in liver hepatocellular carcinoma. Front Pharmacol. 2024;15:1338929.

9.	Yoo W, Choi H, Lee J, Lee Y, Park KC, Noh K. CRHBP, a novel multiple cancer biomarker connected with better prognosis and anti-tumorigenicity. Cancer Cell Int. 2024;24(1):391.

10.	Noh K, Choi H, Jo EH, Yoo W, Park KC. Role of SYT11 in human pan-cancer using comprehensive approaches. Eur J Med Res. 2024;29(1):338.

11.	Tang Z, Li C, Kang B, Gao G, Li C, Zhang Z. GEPIA: a web server for cancer and normal gene expression profiling and interactive analyses. Nucleic Acids Res. 2017;45(W1):W98-W102.

12.	Xie Z, Bailey A, Kuleshov MV, Clarke DJB, Evangelista JE, Jenkins SL, et al. Gene Set Knowledge Discovery with Enrichr. Curr Protoc. 2021;1(3):e90.

13.	Massalha H, Bahar Halpern K, Abu-Gazala S, Jana T, Massasa EE, Moor AE, et al. A single cell atlas of the human liver tumor microenvironment. Mol Syst Biol. 2020;16(12):e9682.

14.	Li T, Fu J, Zeng Z, Cohen D, Li J, Chen Q, et al. TIMER2.0 for analysis of tumor-infiltrating immune cells. Nucleic Acids Res. 2020;48(W1):W509-W14.

15.	Liu Z, Liu L, Weng S, Xu H, Xing Z, Ren Y, et al. BEST: a web application for comprehensive biomarker exploration on large-scale data in solid tumors. Journal of Big Data. 2023;10(1):165.

16.	Yoo W, Kim S, Noh K. SAMD13 serves as a useful prognostic biomarker for hepatocellular carcinoma. Eur J Med Res. 2023;28(1):514.
